# Systemic reserve dysfunction and contrast-associated acute kidney injury following percutaneous coronary intervention

**DOI:** 10.1371/journal.pone.0299899

**Published:** 2024-03-05

**Authors:** Mi-Jeong Kim, Doo Soo Jeon, Youngchul Ahn, Jaeho Byeon, Dongjae Lee, Ik Jun Choi

**Affiliations:** 1 Department of Cardiology, Incheon St. Mary’s Hospital, The Catholic University of Korea, Incheon, Republic of Korea; 2 Department of Cardiology, College of Medicine, The Catholic University of Korea, Seoul, Republic of Korea; 3 Catholic Research Institute for Intractable Cardiovascular Disease, College of Medicine, The Catholic University of Korea, Seoul, Republic of Korea; Showa University: Showa Daigaku, JAPAN

## Abstract

**Background:**

Developing contrast-associated acute kidney injury (CA-AKI) following percutaneous coronary intervention (PCI) is closely related to patient-related risk factors as well as contrast administration. The diagnostic and prognostic roles of neutrophil gelatinase-associated lipocalin (NGAL) in CA-AKI following PCI are not well established.

**Methods:**

Consecutive patients undergoing PCI were enrolled prospectively. CA-AKI was defined as an increase in the serum creatinine level ≥0.3 mg/dL within 48 hours or ≥1.5 times the baseline within 7 days after PCI. Serum NGAL concentrations were determined immediately before and 6 hours after PCI. The participants were classified into four NGAL groups according to the pre- and post-PCI NGAL values at 75th percentile.

**Results:**

CA-AKI occurred in 38 (6.4%) of 590 patients. With chronic kidney disease status (hazard ratio [HR] 1.63, 95% confidence interval [CI]: 1.06–2.52), NGAL groups defined by the combination of pre- and 6 h post-PCI values were independently associated with the occurrence of CA-AKI (HR 1.69, 95% CI: 1.16–2.45). All-cause mortality for 29-month follow-ups was different among NGAL groups (log-rank *p*<0.001). Pre-PCI NGAL levels significantly correlated with baseline cardiac, inflammatory, and renal markers. Although post-PCI NGAL levels increased in patients with larger contrast administration, contrast media made a relatively limited contribution to the development of CA-AKI.

**Conclusion:**

In patients undergoing PCI, the combination of pre- and post-PCI NGAL values may be a useful adjunct to current risk-stratification of CA-AKI and long-term mortality. CA-AKI is likely caused by systemic reserve deficiency rather than contrast administration itself.

## Introduction

Acute kidney injury (AKI) following percutaneous coronary intervention (PCI) is associated with adverse short- and long-term outcomes [1–7]. Patient-related factors affecting physical resilience and contrast administration are critical to the development of contrast-associated acute kidney injury (CA-AKI) [8–14].

Neutrophil gelatinase-associated lipocalin (NGAL) is a marker of acute renal tubular injury and has been introduced as a promising biomarker with temporal advantages over serum creatinine [15,16]. Following PCI-related renal injury, the serum concentration of NGAL increases within a few hours, preceding the decline in the estimated glomerular filtration rate (eGFR) [17,18]. Non-renal diagnoses, including systemic inflammation, atherosclerosis, and myocardial disease, also contribute to NGAL elevation [19–21]. Nevertheless, there is still controversy over the diagnostic and prognostic roles of serum NGAL in CA-AKI following PCI.

We aimed to quantify the respective contribution of patients’ pre-existing vulnerability factors using serum NGAL level before PCI and the contrast-induced renal injury using serum NGAL level after PCI to the development of CA-AKI and long-term mortality.

## Materials and methods

### Study design

This single-center prospective cohort study includes consecutive patients aged >20 who underwent elective or urgent PCI for the first time between September 2015 and November 2017 at Incheon St. Mary’s Hospital. The exclusion criteria comprised cardiogenic shock requiring resuscitation or mechanical left ventricular support, failed PCI, repeated revascularization within one month, an end-stage renal disease requiring maintenance dialysis, the use of nephrotoxic drugs other than contrast dye, organ transplantation, active malignancy, and any moribund condition with an expected survival of less than 1 year.

The primary endpoints were CA-AKI occurrence after index PCI and all-cause mortality during up to 3-year follow-up. The survival data were collected from medical records and the National Health Insurance Corporation through January 31, 2020.

### Ethical statement

The study protocol was approved by the institutional review board (IRB No. OIRB-00247-009) and conformed with the principles of the Declaration of Helsinki. Written informed consent was obtained from all participants in the study.

### Clinical variables

CA-AKI was defined as an increase in the serum creatinine level ≥0.3 mg/dL within 48 hours or ≥1.5 times the baseline within 7 days after PCI [22]. Serum creatinine values were obtained at the baseline as well as at 6 hours and 1, 2, and 7 days after PCI. Day 7 blood samples were obtained from the outpatient clinic for most patients.

Pre-existing comorbidities were identified using renal (eGFR and albuminuria), cardiac (left ventricular ejection fraction [EF] and N-terminal pro-b-type natriuretic peptide [NT-proBNP]), and inflammatory (peripheral blood neutrophil count and high-sensitive C-reactive protein [hs-CRP]) biomarkers. The eGFR was calculated using serum creatinine according to the Chronic Kidney Disease Epidemiology Collaboration equation [23]. Albuminuria was determined using the albumin-creatinine ratio (ACR) in random urine samples. The baseline CKD status was defined by the risk categories of “low risk” (reference), “moderately increased risk,” “high risk,” and “very high-risk” groups by eGFR and urine ACR according to the Kidney Disease Improving Global Outcomes guidelines [24]. Mehran scores were calculated to predict the risk of CA-AKI [9].

### Serum NGAL measurement and NGAL Group definition

Blood samples for NGAL assays were obtained through the arterial sheath before PCI (pre-PCI NGAL) and at 6 hours after PCI (6 h post-PCI NGAL). The samples were immediately centrifuged, and the plasma was stored at -80°C until analysis. The concentration of NGAL was determined with an enzyme-linked immunosorbent assay using a Human Lipocalin-2/NGAL Quantikine ELISA Kit (R&D systems, Minneapolis, MN, USA) according to the manufacturer’s instructions. Intra- and inter-assay variabilities were 4.2% and 9.5%, respectively. All samples were measured in duplicate in a core laboratory (The Clinical Research Laboratory, Incheon St. Mary’s Hospital) by independent and blinded operators.

Serum NGAL values were sorted into quartiles, with values in the highest quartile (Q4) considered “high.” According to pre- and 6 h post-PCI serum NGAL values, we defined four NGAL Groups as following: ≤ the 75th percentile at both pre- and post-PCI (Group 1, “Low-Low”); > the 75th percentile at pre- and ≤ the 75th percentile at post-PCI (Group 2, “High-Low”); ≤ the 75th percentile at pre- and > the 75th percentile at post-PCI (Group 3, “Low-High”); and > the 75th percentile at both pre- and 6 h post-PCI (Group 4, “High-High”).

### PCI procedure

PCI was performed through the radial or femoral artery at the operator’s discretion. All patients were treated with drug-eluting stents or balloon angioplasty and received standard medical therapy according to the prevailing guidelines [25–27]. A preparatory intravenous hydration protocol was used to prevent CA-AKI according to the practice guidelines [25]. Specifically, 0.9% saline was administered from 12 h before 24 h after PCI at the rate of 1 mL/kg/h (0.5 mL/kg/h for patients with left ventricular ejection fraction ≤35% or symptomatic heart failure). For urgent procedures, intravenous hydration was started when the PCI was planned. The iso-osmolar contrast medium Iodixanol (Visipaque; GE Healthcare, Chicago, IL, USA) was used in all patients. Clinical follow-up was performed every 3 months for 1 year and then annually via in-person visits or telephone calls.

### Statistical analysis

Continuous variables were presented as mean ± SD or median (interquartile range), and categorical variables are presented as frequencies and percentages. The Student t-test and Chi-squared test were used to compare patients with and without CA-AKI. For NGAL groups, analysis of variance for continuous variables and the Chi-squared test for categorical variables were performed. For nonparametric variables, including serum NGAL values, the median values of the groups were compared with the Kruskal–Wallis test, and Bonferroni correction was used for multiple comparisons. Linear correlations were measured with the Pearson correlation coefficient. Logistic regression analyses were performed to identify predictors for CA-AKI. Log transformation was used to assess the correlations and regression for nonparametric variables, including NGAL. Receiver operating characteristic curve (ROC) analysis was used to determine the cut-off threshold for the CA-AKI association and NGAL group classification. A Kaplan–Meier plot was used to compare all-cause mortality. Binary logistic regression was performed to identify the independent predictors of the development of CA-AKI, while the Cox proportional hazards regression was performed for long-term mortality. The volume of contrast media per 50 mL associated with CA-AKI occurrence was assessed with logistic regression model fitness (R^2^). A two-sided *p*-value <0.05 indicated statistical significance, and analyses were performed using R version 4.1.1 software (R Foundation for Statistical Computing, Vienna, Austria).

## Results

### Study population, the incidence of CA-AKI, and long-term mortality

**Fig 1:**
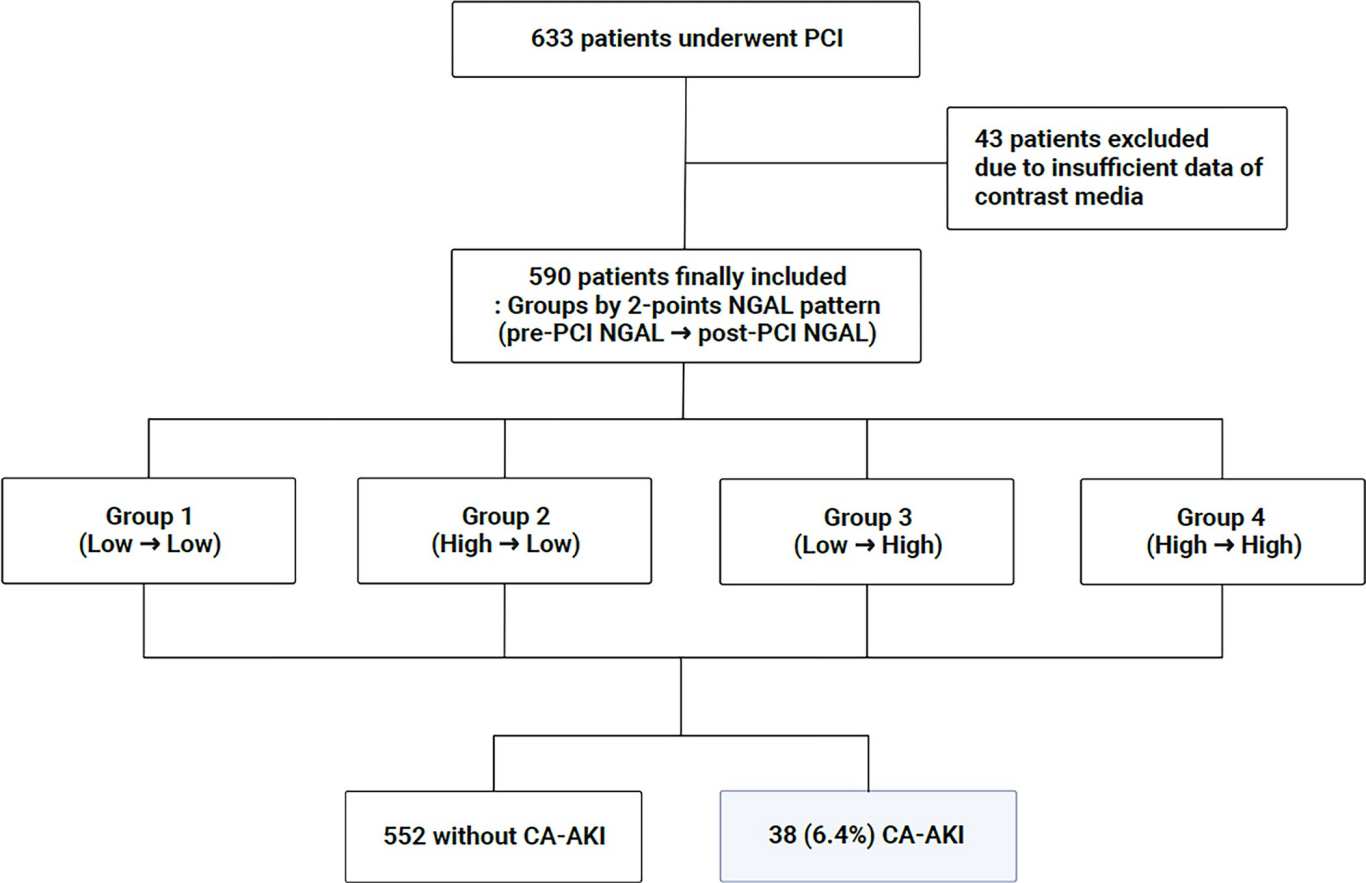
Study flow chart. Patients were classified into four groups based on the pattern of change in the serum NGAL values obtained pre- and post-PCI. CA-AKI, contrast-associated acute kidney injury; NGAL, neutrophil gelatinase-associated lipocalin; PCI, percutaneous coronary intervention.

The study flow chart is presented in Fig 1. The final study population comprised 590 patients (mean age of 65.7 ± 11.7 years, 33.9% females). Following PCI, CA-AKI occurred in 38 (6.4%) patients. Patients with CA-AKI had adverse baseline characteristics such as being older and hypertensive, and having diabetes, higher levels of inflammatory markers, and cardiac and renal dysfunction, compared to those without CA-AKI (S1 Table). During the median follow-up period of 29.2 months, 33 (5.6%) patients died. Patients with CA-AKI demonstrated significantly shorter survival (log-rank *p* <0.001, S1 Fig).

### Post-PCI NGAL: A risk marker of CA-AKI

**Fig 2:**
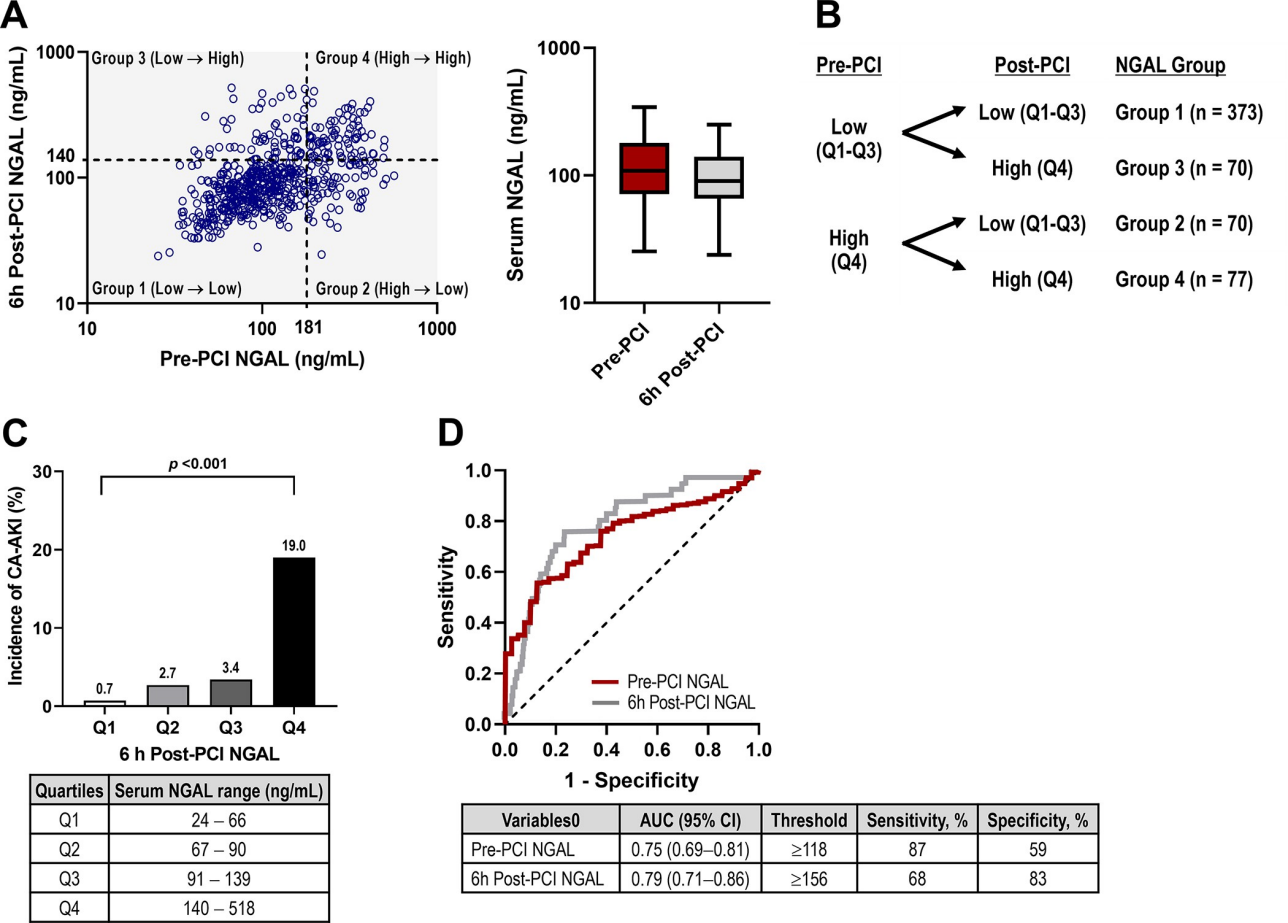
The combination of pre- and 6 h post-PCI serum NGAL values. (A) Distribution of pre- and 6 h post-PCI NGAL values. Cut-off values were determined at 75th percentiles. (B) Serum NGAL group definition according to pre- and post-PCI serum NGAL levels. (C) Post-PCI NGAL quartiles and the incidence of CA-AKI. (D) Receiver operating characteristic curve analysis for the development of CA-AKI. CA-AKI, contrast-associated acute kidney injury; NGAL, neutrophil gelatinase-associated lipocalin; PCI, percutaneous coronary intervention.

The median value of pre- and 6 h post-PCI serum NGAL was 108.6 (71.5–178.2) and 90.4 (65.8–139.2) ng/mL, respectively (Fig 2A). The 75th percentile value was 181 and 140 ng/mL, respectively. The study population was classified into quartiles according to pre- and post-PCI serum NGAL values (Fig 2B). Baseline characteristics according to each group are shown in Table 1.

**Table 1:** Baseline characteristics according to pre- and post-PCI serum NGAL levels.

	Group 1 Low → Low (n = 373)	Group 2 High → Low (n = 70)	Group 3 Low → High (n = 70)	Group 4 High → High (n = 77)	*P*-value
**Age, years**	64.5 ± 11.0	63.6 ± 12.9	66.2 ± 11.8	72.8 ± 11.8^a^	<0.001
**Female**	133 (35.7)	14 (20.0)^a^	27 (38.6)	26 (33.8)	0.065
**Body mass index, kg/m** ^**2**^	24.9 ± 3.3	24.7 ± 3.4	24.6 ± 3.8	24.5 ± 3.6	0.790
**Systolic BP, mmHg**	133 ± 24	134 ± 27	134 ± 23	135 ± 26	0.916
**Heart rate, bpm**	79.2 ± 17.5	79.5 ± 19.7	82.1 ± 17.6	83.1 ± 21.9	0.280
**Previous medical history**					
Hypertension	256 (68.6)	44 (62.9)	53 (75.7)	69 (89.6)^a^	0.001
Diabetes mellitus	124 (33.2)	27 (38.6)	29 (41.4)	47 (61.0)^a^	<0.001
Stroke	31 (8.3)	6 (8.6)	8 (11.4)	16 (20.8)^a^	0.012
PCI/CABG	50 (13.4)	7 (10.0)	12 (17.2)	12 (15.6)	0.622
**Hemoglobin, g/dL**	13.6 ± 1.8	14.1 ± 1.8	13.3 ± 2.0	12.4 ± 2.6^a^	<0.001
**Total cholesterol, mg/dL**	135 ± 34	131 ± 25	141 ± 31	135 ± 32	0.585
**Serum albumin, g/dL**	4.2 ± 0.4	4.1± 0.5	4.0± 0.4	3.8 ± 0.4^a^	<0.001
**Hemoglobin A1C, %**	6.4 ± 1.2	6.7 ± 1.4	6.5 ± 1.1	6.9 ± 1.5^a^	0.024
**Inflammatory markers**					
Neutrophil count, /L	3.9 (2.2)	4.7 (2.2)^b^	4.0 (2.6)	5.9 (2.1)^a^	<0.001
High-sensitive CRP, mg/L	1.1 (2.6)	2.1 (2.7)	1.5 (4.1)	4.3 (30.3)^a^	<0.001
**Cardiac markers**					
Left ventricular EF, %	56.7 ± 10.6	53.8 ± 11.3	53.9 ± 13.5	48.2 ± 15.4^a^	<0.001
NT-proBNP, pg/mL	111 (405)	123 (610)	182 (766)^b^	1,026 (6,213)^a^	<0.001
Troponin T, pg/mL	14 (38)	32 (158)^b^	18 (80)	38 (220)^a^	<0.001
**Renal markers**					
eGFR, mL/min/1.73 m^2^	82.9 ± 18.6	81.5 ± 21.6	73.4 ± 24.8^b^	55.3 ± 26.2^a^	<0.001
ACR, mg/g	9.0 (15.9)	17.0 (33.5)^b^	19.2 (75.3)^b^	71.0 (281.4)^a^	<0.001
**Mehran score**	5.2 ± 4.1	5.9 ± 4.5	7.4 ± 5.5^b^	11.1 ± 6.0^a^	<0.001
**Serum NGAL**					
Pre-PCI, ng/mL	86 (50)	250 (138)^b^	118 (59)^b^	275 (123)^b^^,^^d^	<0.001
6 h post-PCI, ng/mL	77 (37)	91 (43)^b^	198 (128)^b^^,^^c^	208 (93)^b^^,^^c^	<0.001
**Index PCI**					
Acute MI	158 (42.4)	49 (70.0)^a^	27 (38.6)	47 (61.0)^b^^,^^d^^,^	<0.001
Inotropic support	27 (7.2)	7 (10.0)	13 (18.8)^b^	11 (14.3)^b^	0.014
Culprit lesion in LAD	203 (55.6)	33 (47.1)	39 (55.7)	32 (43.2)	0.206
Multivessel disease	109 (29.2)	24 (34.3)	20 (28.6)	36 (46.8)^a^	0.023
Femoral approach	64 (17.2)	12 (17.1)	22 (31.4)^b^	25 (32.5)^b^	0.002
Number of stents	1.7 ± 1.0	1.8 ± 1.1	1.6 ± 0.8	2.1 ± 1.3^a^	0.009
Total length of stents, mm	41.0 ± 29.3	46.3 ± 32.7	39.0 ± 24.3	58.6 ± 41.7^a^	<0.001
Contrast volume, mL	143 ± 64	146 ± 61	162 ± 90^a^	160 ± 70^b^^,^^c^	0.058

Data are mean ± SD, median (interquartile range), or n (%).

^a^ p<0.05 compared to all other groups.

^b^ p<0.05 compared to the reference group (Group 1).

^c^ p<0.05 compared to Group 2.

^d^ p<0.05 compared to Group 3.

ACR, albumin-creatinine ratio; BP, blood pressure; CABG, coronary artery bypass graft; CRP, C-reactive protein; EF, ejection fraction; eGFR, estimated glomerular filtration rate; LAD, left anterior descending; MI, myocardial infarction; NGAL, neutrophil gelatinase-associated lipocalin; NT-proBNP, N-terminal pro-b-type natriuretic peptide; PCI, percutaneous coronary intervention.

The higher post-PCI NGAL values were related to the development of CA-AKI (Fig 2C). The number of patients with CA-AKI in each quartile of 6 h post-PCI NGAL was 1 (0.7%), 4 (2.7%), 5 (3.4%), and 28 (19.0%), respectively (*p* <0.001). Post-PCI NGAL values correlated significantly with baseline eGFR, albuminuria, markers of systemic inflammation and cardiac dysfunction, and the volume of contrast media administrated (S2 Table). Moreover, the post-PCI level tends to depend on the pre-PCI one (r = 0.498, *p* <0.001). Compared to those in Group 1 (reference), the NGAL values of the patients in Group 3 had increased after PCI, despite being in the normal range before the procedure and patients exhibiting higher need for inotropic support, femoral approach, and a larger amount of contrast media (Table 1).

### Pre-PCI NGAL: A summary marker for baseline risk factors

An increased pre-PCI NGAL value was associated with decreased eGFR, albuminuria, systemic inflammation, and abnormal cardiac markers (S2 Table). Patients with the highest quartile of pre-PCI NGAL (Group 2 and Group 4) had more acute MI, an elevated neutrophil count, and microalbuminuria (Table 1). The CA-AKI predictability of pre-PCI NGAL was as good as that of post-PCI NGAL (Fig 2D).

Out of 147 patients who had elevated NGAL levels before PCI (Group 2 and Group 4), 70 had decreased NGAL values after the procedure (Group 2), while 94 did not (Group 4). Group 4 exhibited a significantly higher prevalence of adverse baseline characteristics, including age, comorbidities and systemic inflammation, cardiac and renal dysfunction, and extensive coronary artery diseases, as well as a larger volume of contrast administration, compared to Group 2 (Table 1).

### Risk stratification by the NGAL group defined using the combination of pre- and post-PCI NGAL

**Fig 3:**
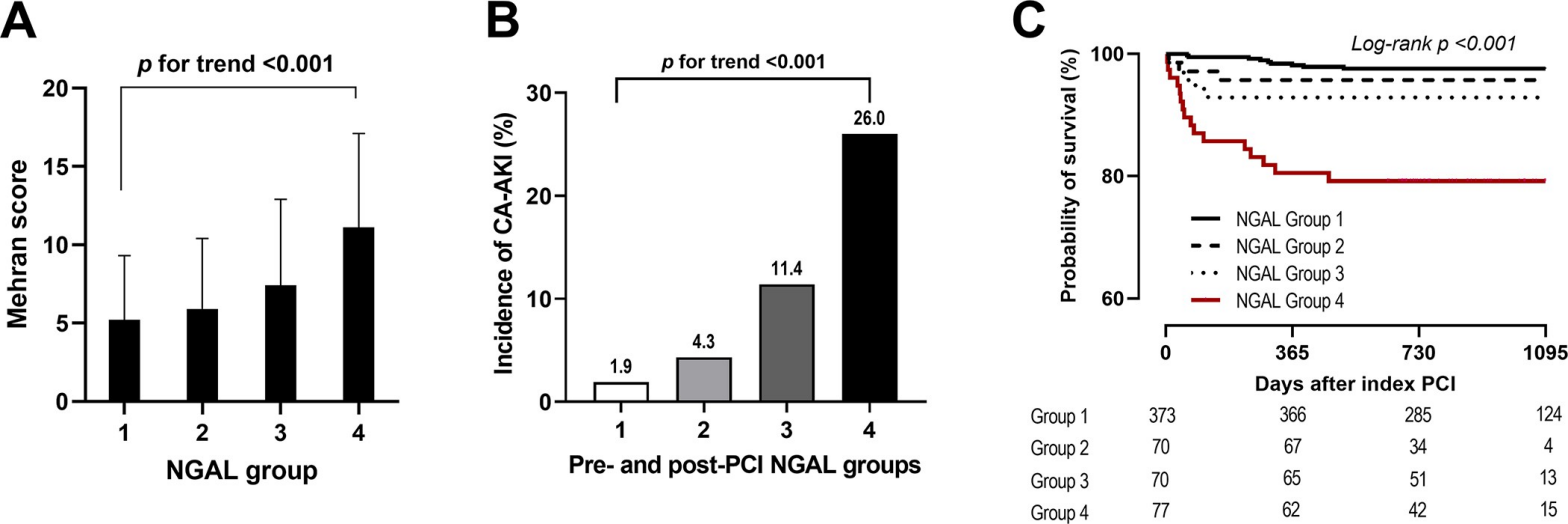
Risk stratification by NGAL groups. (A) Mehran score in NGAL groups. (B) Incidence of CA-AKI in each group. (C) All-cause mortality at long-term follow-up. CA-AKI, contrast-associated acute kidney injury; NGAL, neutrophil gelatinase-associated lipocalin.

Mehran scores were correlated with NGAL groups (Fig 3A). The incidence of CA-AKI differed among the NGAL groups (Fig 3B): 7 (1.9%) cases in Group 1, 3 (4.3%) in Group 2, 8 (11.4%) in Group 3, and 20 (26.0%) in Group 4 (*p*-value for trend <0.001). All-cause mortality during the follow-up period was different among the groups (log-rank *p* <0.001, Fig 3C).

### Relationship between CKD status and NGAL groups

**Fig 4:**
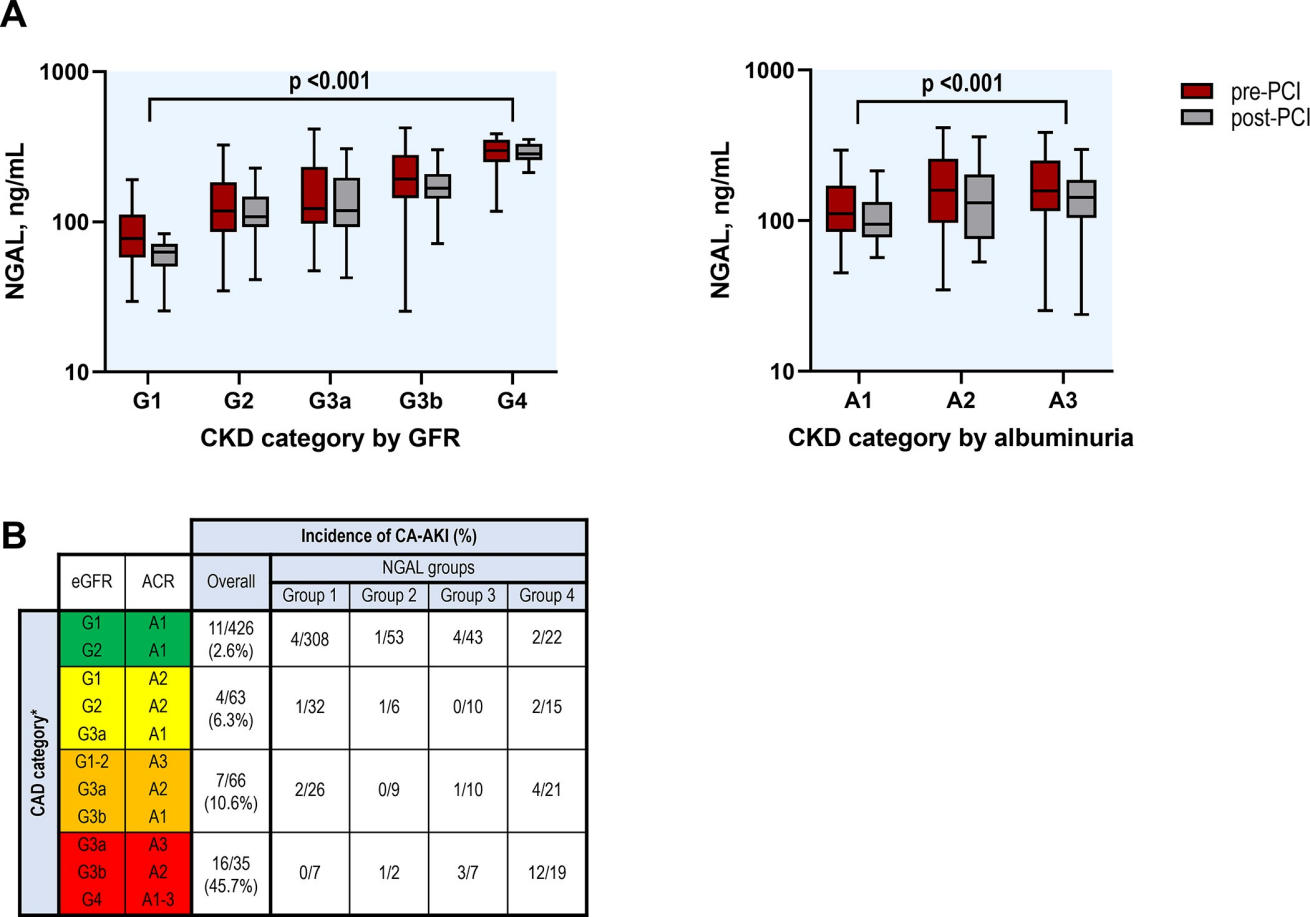
Relationship between NGAL groups and baseline chronic kidney disease (CKD) risk categories. (A) NGAL levels according to CKD risk categories by estimated glomerular filtration rate (eGFR) and albumin-creatinine ratio. (B) Incidence of CA-AKI according to NGAL groups and CKD risk categories. CA-AKI, contrast-associated acute kidney injury; NGAL, neutrophil gelatinase-associated lipocalin.

Baseline CKD risk categories were related to pre- and 6 h post-PCI NGAL levels (Fig 4A). CA-AKI incidence tended to increase with the advance in CKD severity (Fig 4B). In the multivariable analysis, CKD risk categories (HR 1.63, 95% CI: 1.06–2.52) and two-time point NGAL group (HR 1.69, 95% CI: 1.16–2.45) were identified as independent factors of CA-AKI (Table 2). However, with long-term mortality, the baseline CKD risk category was not independently associated, unlike left ventricular EF (HR 1.61, 95% CI: 1.20–2.15) and two-time point NGAL group (HR 1.70, 95% CI: 1.25–2.32), which demonstrated a significant association (Table 3).

**Table 2:** Variables associated with contrast-associated acute kidney injury.

	Univariable		Multivariable	
	HR (95% CI)	*P*-value	HR (95% CI)	*P*-value
**Age (per 10 years)**	1.53 (1.14–2.06)	0.005	0.98 (0.65–1.47)	0.903
**Female sex**	1.15 (0.58–2.27)	0.692	-	-
**Diabetes Mellitus**	3.78 (1.87–7.66)	<0.001	-	-
**Neutrophil count (decile)**	1.30 (1.14–1.49)	<0.001	-	-
**Left ventricular EF (per 10%)**	2.40 (1.81–3.17)	<0.001	1.46 (0.94–2.28)	0.092
**CKD risk category**	2.98 (2.21–4.01)	<0.001	1.61 (1.05–2.48)	0.027
**Mehran score**	1.25 (1.17–1.33)	<0.001	1.07 (0.94–1.22)	0.312
**Contrast volume (per 50 mL)**	0.97 (0.75–1.25)	0.814	0.92 (0.68–1.27)	0.624
**Pre-PCI NGAL (decile)**	1.40 (1.21–1.61)	<0.001	-	-
**NGAL group**	2.66 (1.98–3.57)	<0.001	1.69 (1.18–2.44)	0.005

CI, confidence interval; CKD, chronic kidney disease; EF, ejection fraction; HR, hazard ratio; NGAL, neutrophil gelatinase-associated lipocalin; PCI, percutaneous coronary intervention.

**Table 3:** Variables associated with long-term all-cause mortality.

	Univariable		Multivariable	
	HR (95% CI)	*P*-value	HR (95% CI)	*P*-value
**Age (per 10 years)**	2.94 (1.98–4.38)	<0.001	2.30 (1.49–3.55)	<0.001
**Female sex**	3.09 (1.54–6.20)	0.002	2.09 (0.98–4.42)	0.055
**Diabetes Mellitus**	2.23 (1.12–4.44)	0.023	1.68 (0.81–3.48)	1.165
**Neutrophil count (decile)**	1.52 (1.28–1.79)	<0.001	-	-
**Left ventricular EF (per 10%)**	1.84 (1.40–2.42)	<0.001	1.61 (1.20–2.15)	0.001
**CKD risk category**	2.02 (1.53–2.67)	<0.001	0.91 (0.62–1.32)	0.610
**Pre-PCI NGAL (decile)**	1.31 (1.14–1.51)	<0.001	-	-
**NGAL group**	2.13 (1.62–2.81)	<0.001	1.70 (1.25–2.32)	0.001

CI, confidence interval; CKD, chronic kidney disease; EF, ejection fraction; HR, hazard ratio; NGAL, neutrophil gelatinase-associated lipocalin; PCI, percutaneous coronary intervention.

### Role of contrast administration

**Fig 5:**
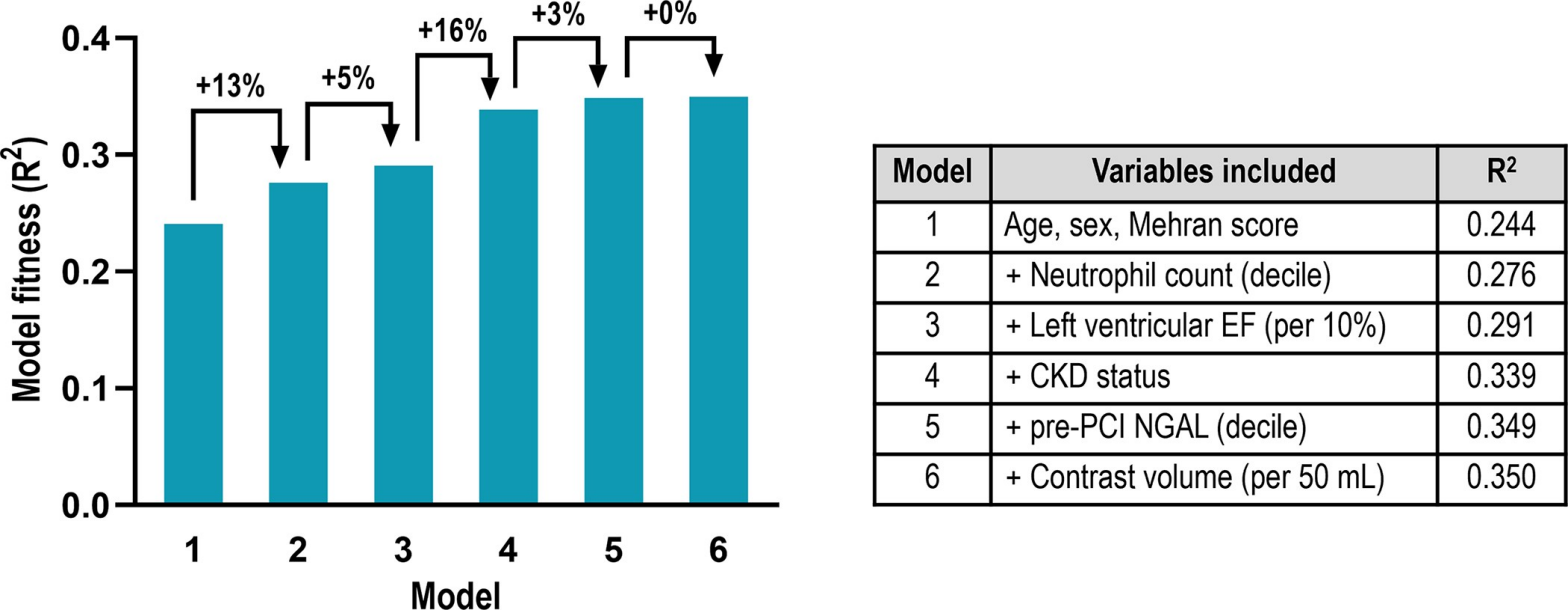
The regression model fitness (R^2^) according to the serial addition of explanatory variables associated with the development of CA-AKI following PCI. CA-AKI, contrast-associated acute kidney injury; PCI, percutaneous coronary intervention.

Although the amount of contrast media used in patients in Group 3 and Group 4 was large compared to that in Group 1 (Table 1), contrast volume was not an independent factor for the development of CA-AKI (Table 2) or long-term mortality (Table 3). In the logistic regression analysis, model fitness (R^2^) for the association of CA-AKI showed a progressive increase as baseline patient-related risk factors were added, including demographics, systemic comorbidities reflected by Mehran score, neutrophil count, left ventricular EF, CKD status, and pre-PCI NGAL value (Fig 5). Conversely, the addition of contrast media did not significantly contribute to model fitness.

## Discussion

We sought to quantify the complex baseline patient-related risk factors with a single-term serum NGAL value. While 6 h post-PCI NGAL levels predicted CA-AKI, which was directly related to index PCI, pre-PCI NGAL levels reflected the severity of underlying cardiac or renal disease. The incidence of CA-AKI and mid-term mortality increased significantly only when both pre- and post-PCI NGAL values were high. Unless serum NGAL increased both before and after PCI, the risk was low. Although several PCI trials have investigated the role of NGAL in predicting CA-AKI or forecasting prognosis, the present study clarified the practical utility of NGAL in an actionable manner.

Systemic frailty of the patient plays a critical role in developing CA-AKI following PCI [28,29]. Endothelial dysfunction is involved across the whole course of atherosclerosis [30,31], resulting in systemic microvascular dysfunction [32]. Patients with advanced coronary artery disease are likely to have additional extracardiac microvascular dysfunction, such as microalbuminuria, which is associated with frailty [33]. In the context of cardiorenal syndrome, localized cardiac events such as myocardial ischemia could evolve into full-blown systemic multiorgan illnesses such as CA-AKI in frail patients, as the body’s resilience is impaired. In the case of myocardial infarction, baseline NGAL is frequently elevated [34] owing to atherosclerotic plaque rupture, myocardial ischemia, systemic leukocyte activation, or CKD, as well as acute renal tubular injury [19–21]. Elevated serum NGAL level indicates complex systemic illness [35–37], which may serve as an alarm sign in patients with coronary artery disease in terms of CA-AKI and long-term mortality. In this study, the NGAL groups defined by the combination of pre- and post-PCI were correlated with the Mehran score. Our strategy using pre- and post-PCI NGAL measurements to summarize the patient-side risk may not be superior to Mehran’s scoring in terms of predicting CA-AKI. However, the NGAL-based grouping is a novel approach to summarizing the patient’s frailty. It would be meaningful as a biomarker reflecting the elaborately calculated Mehran score and a useful complementary tool for predicting CA-AKI.

Our results were consistent with those of the previous reports that investigated the utility of serum NGAL in patients who underwent PCI. In a study including 458 CKD patients undergoing scheduled coronary or peripheral angiography, low serum NGAL levels (<179 ng/mL) at 6 hours after contrast dye exposure were a reliable marker for ruling out CA-AKI, whereas high NGAL levels (≥179 ng/mL) could predict 1-year adverse cardiac or renal events [18]. While the median serum creatinine value was approximately 2.0 mg/dL and 46% of the population had undergone the elective PCI, our study population comprised consecutive patients who underwent elective or urgent PCI, covering the entire range of renal functions, thus being more appropriate for generalizing.

Regarding the optimal cut-off value of NGAL, it is difficult to directly apply the value used in previous reports because the study population is different, and the measurement method is not standardized. Even considering the different causes and diagnostic criteria of AKI, study population, and study endpoints, the meaningful NGAL cutoffs vary greatly depending on the studies [38]. Although the 75th percentile cut-off value used in the present study was arbitrary, we thought it would be more reasonable to select an appropriate upper fraction rather than a specific value to define a high-risk group.

We guessed contrast media is less likely to be a key player in developing CA-AKI based on the literature [12–14] and our results after the adjustment with baseline vulnerability by combining pre- and post- PCI NGAL levels. Because old-generation contrast media with high osmolality are no longer used at present, the classic contrast-induced form of AKI is rare. However, patients with advanced CKD with a bare renal reserve are vulnerable to contrast administration because the filtered contrast material per unit functioning nephron may be highly concentrated if proper hydration is not provided in cases of urgent PCI or decompensated heart failure [39]. The occurrence of AKI in patients undergoing PCI is predominantly caused by patient-related conditions rather than CM administration if an iso-osmolar CM is employed along with contemporary hydration protocols.

The present study has several limitations. The study population was small. Subsequently, only a small number of patients with advanced CKD were included. Considering the low incidence of CA-AKI in current practice, our results cannot be directly generalized in the population with CA-AKI following PCI. However, the 6.4% incidence of CA-AKI in this study was close to 7.1% observed in the multicenter registry, including 985,737 patients [5]. In addition, we defined AKI using serum creatinine; however, serum creatinine is not sensitive or specific to AKI regarding hemodynamic instability, small or ongoing loss of muscle mass (such as in elderly females), advanced frailty or comorbidities, and fluid-loading patients due to dilution [40]. Finally, the exact sources of NGAL were not differentiated in this study. Nevertheless, we selected serum NGAL among renal injury markers for two reasons: firstly, it was the best available representative of patient-related vulnerability, and secondly, to ensure consistent post-PCI sampling timing (i.e., 6 h after PCI). Since the damage induced by contrast media is transient and NGALs were measured both before and after the procedure, our original hypothesis may remain valid. We believe we could appropriately assess renal injury using pre-PCI NGAL to represent baseline vulnerability and post-PCI delta change to estimate additional renal injury.

## Conclusion

In patients undergoing PCI, the combination of pre- and post-PCI serum NGAL values may be a useful adjunct to risk-stratification of CA-AKI and long-term mortality. CA-AKI is likely caused by systemic reserve deficiency due to multiorgan frailty rather than contrast administration *per se*.

## Supporting information

S1 Fig Kaplan–Meier survival estimates for all-cause death after index percutaneous coronary interventions (PCI) clustered by the presence of contrast-associated acute kidney injury (CA-AKI). (TIF)

S1 Table Baseline characteristics in patients with and without CA-AKI after PCI. (DOCX)

S2 Table Bivariate correlation between serum neutrophil gelatinase-associated lipocalin (NGAL) concentrations and systemic markers. (DOCX)
