# Supplementary figures and images for: Systemic reserve dysfunction and contrast-associated acute kidney injury following percutaneous coronary intervention
Source: PLoS One. 2024 Mar 5;19(3):e0299899. doi: 10.1371/journal.pone.0299899 (PMC10914285; doi:10.1371/journal.pone.0299899)

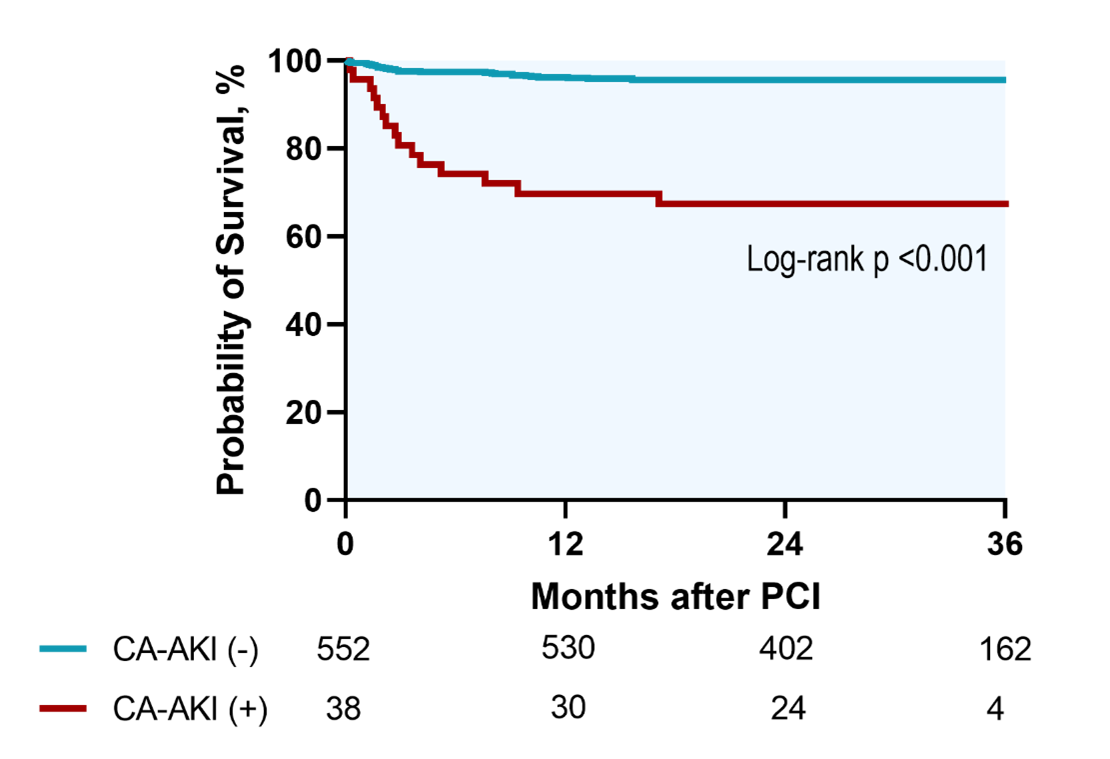

Supplement: S1 Fig — (TIF) [file pone.0299899.s001.tif]
