# Supplementary material for: Systemic reserve dysfunction and contrast-associated acute kidney injury following percutaneous coronary intervention
Source: PLoS One. 2024 Mar 5;19(3):e0299899. doi: 10.1371/journal.pone.0299899 (PMC10914285; doi:10.1371/journal.pone.0299899)
Supplement: S1 Table — (DOCX) [file pone.0299899.s002.docx]

**S1 Table.** Baseline characteristics in patients with and without CA-AKI after percutaneous coronary interventions.

|  | Without CA-AKI  (n = 552) | With CA-AKI  (n = 38) | p-value |
| --- | --- | --- | --- |
| Age (years) | 65.3 ± 11.7 | 71.4 ± 11.0 | 0.002 |
| Female | 186 (33.7) | 14 (36.8) | 0.692 |
| Body mass index (kg/m^2^) | 24.8 ± 3.3 | 24.4 ± 4.4 | 0.593 |
| Systolic blood pressure (mmHg) | 133.3 ± 24.1 | 138.4 ± 26.6 | 0.207 |
| Heart rate (bpm) | 79.7 ± 18.3 | 85.6 ± 19.0 | 0.057 |
| Previous medical history |  |  |  |
| Hypertension | 385 (69.7) | 37 (97.4) | <0.001 |
| Diabetes mellitus | 201 (36.4) | 26 (68.4) | <0.001 |
| Stroke | 54 (9.8) | 7 (18.4) | 0.091 |
| PCI/CABG | 77 (13.9) | 4 (10.5) | 0.612 |
| Hb (g/dL) | 13.5 ± 1.9 | 11.8 ± 2.3 | <0.001 |
| Total cholesterol (mg/dL) | 136.2 ± 32.6 | 123.1 ± 26.7 | 0.073 |
| Serum albumin (g/dL) | 4.1 ± 0.4 | 3.8 ± 0.5 | <0.001 |
| Hemoglobin A1c (%) | 6.5 ± 1.3 | 6.9 ± 1.3 | 0.058 |
| Inflammatory markers |  |  |  |
| Neutrophil count (/L) | 5.2 ± 2.7 | 7.0 ± 3.0 | <0.001 |
| High-sensitive CRP (mg/L) | 1.3 (0.6–12.6) | 2.2 (0.7–33.6) | 0.018 |
| Cardiac markers |  |  |  |
| NT-proBNP (pg/mL) | 136 (46–558) | 3547 (267–8271) | <0.001 |
| Troponin T (pg/mL) | 15.0 (8–58) | 48.5 (23–289) | 0.002 |
| Renal markers |  |  |  |
| eGFR (mL/min per 1.73 m^2^) | 79.7 ± 21.5 | 53.1 ± 26.5 | <0.001 |
| Urine ACR (mg/g) | 10.4 (5–29) | 96.7 (28–450) | <0.001 |
| Serum NGAL |  |  |  |
| Pre-PCI (ng/mL) | 97.5 (67–145) | 149.9 (120–247) | <0.001 |
| Post-PCI (ng/mL) | 86.8 (65–124) | 183.4 (98–222) | <0.001 |
| Left ventricular EF (%) | 55.8 ± 11.3 | 42.5 ± 15.8 | <0.001 |
| Index PCI characteristics |  |  |  |
| Acute MI | 262 (47.5) | 19 (50) | 0.762 |
| Culprit lesion in LAD | 285 (52.2) | 22 (59.5) | 0.392 |
| Multivessel CAD | 173 (31.3) | 16 (42.1) | 0.169 |
| Hemodynamic instability | 56 (10.1) | 4 (10.5) | 0.940 |
| Total of stented length (mm) | 42.4 ± 30.4 | 61.3 ± 42.4 | <0.001 |
| Contrast volume (mL) | 216.8 ± 121.6 | 239.3 ± 95.9 | 0.265 |

Data are mean ± SD, median (IQR), or n (%).

ACR, albumin-creatinine ratio; CAD, coronary artery disease; CABG, coronary artery bypass graft; EF, ejection fraction; eGFR, estimated glomerular filtration rate; CRP, C-reactive protein; LAD, left anterior descending; MI, myocardial infarction; NGAL, neutrophil gelatinase-associated lipocalin; NT-proBNP, N-terminal prohormone B-type natriuretic peptide; PCI, percutaneous coronary intervention.
