# Supplementary material for: Systemic reserve dysfunction and contrast-associated acute kidney injury following percutaneous coronary intervention
Source: PLoS One. 2024 Mar 5;19(3):e0299899. doi: 10.1371/journal.pone.0299899 (PMC10914285; doi:10.1371/journal.pone.0299899)
Supplement: S2 Table — (DOCX) [file pone.0299899.s003.docx]

**S2 Table.** Bivariate correlation between serum NGAL concentrations and systemic markers.

|  | Pre-PCI NGAL | | Post-PCI NGAL | |
| --- | --- | --- | --- | --- |
|  | r | p-value | r | p-value |
| High-sensitive CRP | 0.208 | <0.001 | 0.239 | <0.001 |
| Neutrophil count | 0.355 | <0.001 | 0.306 | <0.001 |
| NT-proBNP | 0.240 | <0.001 | 0.309 | <0.001 |
| Troponin T | 0.253 | <0.001 | 0.186 | <0.001 |
| eGFR | -0.314 | <0.001 | -0.374 | <0.001 |
| Urine ACR | 0.237 | <0.001 | 0.281 | <0.001 |
| Contrast volume, mL | NA | NA | 0.122 | 0.004 |

ACR, albumin-creatinine ratio; eGFR, estimated glomerular filtration rate; CRP, C-reactive protein; NA, not available; NGAL, neutrophil gelatinase-associated lipocalin; NT-proBNP, N-terminal pro-B-type natriuretic peptide; PCI, percutaneous coronary intervention.
